# *Bacillus nematocida* B16 Enhanced the Rhizosphere Colonization of *Pochonia chlamydosporia* ZK7 and Controlled the Efficacy of the Root-Knot Nematode *Meloidogyne incognita*

**DOI:** 10.3390/microorganisms10020218

**Published:** 2022-01-20

**Authors:** Tingting Bo, Chuixu Kong, Shunxing Zou, Minghe Mo, Yajun Liu

**Affiliations:** 1 State Key Laboratory for Conservation and Utilization of Bio-Resources, Yunnan University, Kunming 650032, China; BTT0627@163.com (T.B.); KCX2430@163.com (C.K.); qq420633716@126.com (S.Z.); minghemo@163.com (M.M.); 2 Key Laboratory for Southwest Microbial Diversity of the Ministry of Education, Yunnan University, Kunming 650032, China

**Keywords:** *P. chlamydosporia* ZK7, *B. nematocida* B16, *Meloidogyne incognita*, combination control, volatiles

## Abstract

*Pochonia chlamydosporia* is widely applied in many countries as a biocontrol fungus against parasitic nematodes in plants. In a field experiment, the combined use of *Bacillus nematocida* B16 increased the biocontrol efficiency of *P. chlamydosporia* ZK7 against *Meloidogyne incognita*. Further study indicated that the colonization of *P. chlamydosporia* ZK7 in the rhizosphere soil and the roots of tomatoes was significantly higher in the combined use group than in the control group. Gas chromatography was conducted to determine the effects of signaling substances. Five compounds, hexanal, (E)-2-hexenal, furfural, benzaldehyde, and 2-nonanone, were found to be highly altered in the volatile compounds produced in the soil under the combined application. The changes in benzaldehyde and 2-nonanone were the main factors that resulted in an increase in the colonization of fungi *P. chlamydosporia* ZK7 in the tomato roots. Furfural was the main volatile substance that affected the colonization of fungi *P. chlamydosporia* ZK7 in the soil. The combined use of *B. nematocida* B16 and *P. chlamydosporia* ZK7 altered the volatile ranges and resulted in increased colonization of biocontrol fungi and improved biocontrol efficiency against nematodes. This combined model could be used to promote the ability of biocontrol fungi to control root-knot nematodes.

## 1. Introduction

Root-knot nematodes are a rhizosphere pest and are estimated to cause more than USD 1.73 billion in annual crop loss [1]. These parasitic nematodes not only cause direct damage to plant roots but also encourage other plant pathogens, including fungi, bacteria, and viruses, to infect plant roots. Traditional methods of controlling root-knot nematodes mainly use chemical insecticides. However, large amounts of chemical nematicides not only cause serious environmental problems but also harm human health [2,3]. As a result, many chemical pesticides have been banned or restricted.

*Pochonia chlamydosporia* is widely applied in many countries as a biocontrol fungus against parasitic nematodes in plants [4]. Regarded as one of the most versatile biocontrol agents for nematodes, this antagonist has many desirable properties as a biocontrol fungus. Due to its saprophytic activity, this fungus can survive in the soil even in the absence of nematodes [5]. The primary mechanism of action is through hyphae that invade nematode eggs and female cysts for asexual reproduction and extend out of the body to produce conidia, which leads to the death of eggs and female cysts to help control root-knot nematodes [6]. *Bacillus* is a kind of biocontrol bacterium that is widely distributed in the rhizospheres of plants. It can produce spores that are resistant to stress and can colonize and reproduce well in the soil. Moreover, the volatiles produced by *B. nematocida* B16 have different effects, including inducing plant resistance and nematicide activity [7,8]. However, soil treated with a single biocontrol agent is easily affected by ecological factors, and biocontrol efficiency is often unstable [9]. It is of potential value to use virulence factors of various biocontrol bacteria and their interactions to increase the reliability of nematode biocontrol efficiency [10]. The development of biocontrol agents for nematodes with high efficiency and reliability is much needed.

Microbes interact with each other through chemical-signaling substances. Microbial volatiles have strong diffusion and are often regarded as chemical-signaling molecules in many life activities [11]. Cao [12] found that actinomycete, *Aguyces allii* 130935, had an 89% nematocidal activity against *M. incognita* after 8 h and significantly reduced the root galls. Sphingosine was isolated from *Bacillus cereus* S2. The semi-lethal concentration of sphingosine on *M. incognita* was 0.64 μg/mL [13]. Zhou found that *B. megaterium* Sneb207 inhibits the invasion and reproduction of *Heterodera glycines* by inducing systemic resistance [14]. Two macrocyclic trichothecenes, verrucarin A and roridin A, were isolated from *Meloidogyne verrucaria*. The median effective concentrations of verructin A and roridin A against M. incognita were 1.88 μg/mL and 1.50 μg/mL, respectively [15]. *S. jietaisiensis* could prevent root-knot disease and promote plant growth [16]. Fungi and bacteria are two distinct types of microbes, but they live in close proximity in the same environment and they each recognize the signals that the other produces, causing changes in the expression of related genes and corresponding changes in their behaviors [17]. There have been many reports about the impact of the volatiles produced by bacteria on the growth of fungi. *Streptomyces globisporus* can inhibit the growth of the spore-germ tubes of *Penicillium italicum* and cause abnormal conidia and hyphal shapes [18]. The volatiles of *S. coccidioides* can change the internal structure of the hyphae and the spores of *Botrytis cinerea*. After treatment with volatiles, the hyphae undergo plasmolysis, the cell walls of the spores thicken, and the vacuoles significantly increase [19]. Hassan [20] found that the volatiles of *Bacillus licheniformis* 350-2 had a significant inhibitory effect on the growth, sporulation, and accumulation of the mycotoxins of *Aspergillus flavus* and *Penicillium*.

In previous studies, the combined use of *P. chlamydosporia* ZK7 and *B. nematocida* B16 significantly improved the control effect of *P. chlamydosporia* ZK7 on the root-knot nematode *Meloidogyne incognita*. However, as *B. nematocida* B16 did not show an obvious biocontrol efficiency, this result suggested that the addition of *B. nematocida* B16 influenced the efficiency of the nematode’s colonization ability, which is one of the key biocontrol factors for nematodes under the control of *P. chlamydosporia* ZK7. Thus, the current study investigated changes in chemical-signaling volatiles that result from the combined use of bacteria and fungi in the colonization of *P. chlamydosporia* ZK7, thereby improving its biocontrol ability against nematodes.

## 2. Materials and Methods

### 2.1. Preparation of Strains

Both *P. chlamydosporia* ZK7 and *B. nematocida* B16 were provided and stored by the Culture Collection of the State Key Laboratory for Conservation and Utilization of Bio-Resources in Yunnan. Activated *P. chlamydosporia* ZK7 was incubated in a triangle flask with 250 mL of potato-dextrose-broth (PDB) medium for 72 h to obtain a spore count of 10^6^/mL. The *P. chlamydosporia* ZK7 fermentation broth was filtered with six layers of sterilized microscopic paper to remove the hyphae. The spores were counted using a hemocytometer, and the final concentration of spores was adjusted to 10^4^/mL and stored at 4 °C. The bacterial cells were incubated in a triangular flask with 200 mL of nutrient-broth (NB) medium for 36 h. The optical density of the sample measured at a wavelength of 600 nm (OD600) was 1.8–2.0, and the sample was stored at 4 °C.

### 2.2. Preparation of Meloidogyne incognita and Tomato Seedlings

*Meloidogyne incognita* eggs were isolated from the root knots of tomato plants cultivated in a greenhouse, and eggs were collected on the 45th day after inoculation. The tomato roots were washed with a large amount of egg suspension, placed in a 500 mL triangular flask with 200 mL of 1% NaClO, and shaken vigorously for 3 min. The egg suspension was rinsed and collected in a 400-mesh sieve. The concentration was adjusted to 200 granules/mL, and the samples were stored at 4 °C.

Tomato seeds (Zhong za No.9, produced by Vegetable and Flower Research Institute, Chinese Academy of Agricultural Sciences) were disinfected with 5% NaClO for 5 min and washed 3–5 times with sterile water. The seeds were transferred to a 16 cm culture dish with a layer of filter paper and incubated at a constant temperature of 28 °C for 3–4 days. After germination, the seeds were transferred to a drift tray with the substrate, incubated in a light incubator for 21 days (temperature of 28 °C and 75% humidity), and prepared for use after 4–6 new leaves had grown.

### 2.3. Field Experiments

The field experiment was conducted in Tong Hai County, Yunnan Province. The fermentation broth of strain *P. chlamydosporia* ZK7 was mixed with the fermentation broth of strain *B. nematocida* B16 in different proportions (i.e., 1:7, 1:3, 1:2, *P. chlamydosporia* ZK7 alone, *B. nematocida* B16 alone). For each treatment, 100 mL of a mixture of bacterial supernatant and spore suspension was added to each plant. The positive control was 100 mL of 0.5% avermectin per plant. The negative control was a blank control wherein no agents were used. All agents were applied at the time of transplantation, with 15 tomato plants for each treatment, and each treatment was repeated 3 times and randomly arranged. The control effect of each treatment was calculated. After 30 and 60 days of tomato transplantation, five tomatoes were collected for each treatment, and the following parameters were measured: the plant height, which is the distance from the soil to the top of the plant, and the fresh weight of the aboveground parts of the plant, which was measured using a balance after uprooting the plants, washing the roots under tap water, and cutting off the underground part with scissors. At 30, 60, and 90 days after transplantation, 5 tomatoes from each treatment were selected to investigate the disease index and biocontrol efficiency according to the classification method [21]: 0, no galls; I, 1–24% of the root galled; II, 25–49% of the root galled; III, 50–74% of the root galled; IV, 75–99% of the root galled; and V, all the roots galled, wherein:

$$\mathrm{disease}\;\mathrm{index}=\frac{\sum (\mathrm{number}\;\mathrm{of}\;\mathrm{plants}\;\mathrm{al}\;\mathrm{all}\;\mathrm{levels}\times \mathrm{series})}{\left(\mathrm{total}\;\mathrm{number}\;\mathrm{of}\;\mathrm{plants}\;\mathrm{surveyed}\times 5\right)}\times 100\%;$$

$$\mathrm{biocontrol}\;\mathrm{efficiency}\;\left(\%\right)=\frac{\left(\mathrm{control}\;\mathrm{disease}\;\mathrm{index}-\mathrm{treatment}\;\mathrm{disease}\;\mathrm{index}\right)}{\mathrm{control}\;\mathrm{disease}\;\mathrm{index}}\times 100\%.$$

### 2.4. Germination of Fungal Spores of P. chlamydosporia ZK7

The *B. nematocida* B16 supernatant was mixed with the *P. chlamydosporia* ZK7 fermentation broth at proportions of 1:1, 1:3, 1:5, 1:7, and 1:10 and incubated on a rotary shaker at 28 °C and 170 rpm. Nutrient-broth medium and *P. chlamydosporia* ZK7 fermentation broth were used as controls. The spores of each treatment and control were counted using a hemocytometer after 4 h, 8 h, 12 h, and 24 h, and each treatment was conducted in triplicate.

### 2.5. Egg Hatching of Meloidogyne incognita

Sterile water (2 mL) and 1 mL egg suspension were added to a sterilized culture dish with a diameter of 60 mm, and then a 1 mL mixture of *B. nematocida* B16 supernatant and *P. chlamydosporia* ZK7 spore suspension (volume ratio: 1:7) was added. Sterile water was used as the control. The culture dishes were sealed with paraffin. Three replicates were performed for each treatment and control. The culture was placed in an incubator at 25 °C for 7–8 d. The number of hatched nematodes was counted under an optical microscope (10 × 10), and the relative inhibition rate was calculated.

### 2.6. Analysis of Volatiles by Gas Chromatography–Mass Spectrometry

To detect volatiles in the soil sample, 3 g of the pot experiment soil and 3 mL of sterile deionized water were added to a 15 mL headspace sample bottle. The bottle was corked, sealed with parafilm, and balanced at 20–25 °C for 5 days. The SAAB-57318 75 μm CAR/PDMS SPEM fiber was inserted and exposed approximately 1.5 cm above the liquid and stirred at 65 °C for 1 h. After sample extraction, a needle was inserted into the sample inlet of the gas chromatography gasification chamber, the fiber head was gently pushed downward, and the high temperature and heat conditions of the gasification chamber were used to allow the substance to be analyzed for 1 min for detection and analysis. Volatile organic compounds (VOCs) were detected by gas chromatography–mass spectrometry (GC/MS) using an Agilent 7890 GC/5975 MSD instrument. The column temperature was initially held at 50 °C for 2 min, raised to 180 °C at a rate of 6 °C per min, and then increased to 240 °C at 8 °C per min, with a final hold time of 10 min. Helium at a linear velocity of 1.0 mL/min was used as the carrier gas. Compounds were identified by comparing the mass spectra with synthetic compounds and database data (NIST111L).

### 2.7. Real-Time Polymerase Chain Reaction of P. chlamydosporia ZK7

Genomic DNA of *P. chlamydosporia* ZK7 was extracted [22], amplified, and purified by polymerase chain reaction (PCR) (Takara DNA Purification Recovery Kit). Specific primers, namely, Spc-F1 (5′ CGTTTCCAGTACaAGA 3′) and SPC-R2 (5′ TCTTCCTCTCAGTTGCCG 3′), these being fragments derived from the VCP1 gene, were used, with a total length of 136 bp [18]. The purified PCR product was connected and transformed into *Escherichia coli* DH5α-competent cells and coated on an LB-ampicillin (AMP) plate. Single bacterial colonies were selected for colony PCR, and the colonies corresponding to the band size (136 bp) were selected and inoculated into LB-AMP liquid medium. The bacterial liquid was sent to BGI (Beijing Genomics institution) for sequencing verification. After the colony PCR-sequencing results were verified, the plasmid was extracted with a high-purity plasmid small-amount preparation kit. The high-purity (OD260/OD280 = 1.8~2.0) positive clone plasmid to multiple dilutions (10× Ã–) was selected, and the final concentration was 4.3 × 102, 103, 104, 105, 106, 107, and 108. The standard curve was prepared by quantitative PCR after dilution of the standard substance. The HP Fungal DNA Kit (OMEGA) was used to extract the DNA of the fungus in the root and the *P. chlamydosporia* ZK7 genomic DNA was stored at −20 °C. The *P. chlamydosporia* ZK7 genome DNA was extracted from the soil according to the instructions of the Soil DNA Kit (OMEGA). After the extraction was completed, the DNA was saved at −20 °C for later use. The reaction conditions and system were the same as those for the preparation of the *P. chlamydosporia* ZK7 standard curve; however, the template was replaced with soil DNA and root fungal DNA.

### 2.8. Pot Experiments

Hexanal, (E)-2-hexenal, furfural, benzaldehyde, and 2-nonanone were added to 100 mL of *P. chlamydosporia* ZK7 fermentation broth and fully stirred to the final concentrations of 200 ppm, 400 ppm, and 800 ppm. The blank control consisted of 100 mL of *P. chlamydosporia* ZK7 fermentation broth. Fermentation broth was added to the potted soil, and tomato seedlings with consistent growth and no disease were selected and transplanted into pots. After 30 days of transplanting, 0.2 g root and 60 g soil samples were collected to detect the colonization of *P. chlamydosporia* ZK7. The DNA of the root fungi was extracted with an HP Fungal DNA Kit (OMEGA), and the extraction procedure of the *P. chlamydosporia* ZK7 genome in the soil was performed according to the instructions of the Soil DNA Kit (OMEGA). After the extraction was completed, the samples were stored at −20 °C. The reaction conditions and system were the same as those of the *P. chlamydosporia* ZK7 standard curve, and the templates were replaced by soil DNA and root fungal DNA, respectively.

The experimental soil samples were evenly mixed according to the ratio of field soil-humus soil-vermiculite at 3:1:1, and tomato seedlings with 4–6 leaves, consistent growth, and no disease were transplanted into 15 cm diameter pots filled with mixed soil samples. Two treatments were designed according to the efficiency of the field experiments performed earlier. Fermentation broth (100 mL) of *B. nematocida* B16-*P. chlamydosporia* ZK7 (1:7, *v*/*v*) was added around the root, and 100 mL of fermentation broth *P. chlamydosporia* ZK7 was used as a control. Each treatment was arranged in 15 pots, and 3 replicates were performed. The biocontrol efficiency and colonization of *P. chlamydosporia* ZK7 were detected at 0, 15, 30, 45, and 60 days.

### 2.9. Statistical Analysis

For the different biocontrol efficiency indexes, e.g., germination of fungal spores and relative inhibition rates of eggs by *P. chlamydosporia* ZK7, the data are shown as the mean ± standard deviation (*n* ≥ 3). Comparisons of significance were performed using one-way analysis of variance (ANOVA) and Tukey’s multiple comparison test at *p* < 0.05. All statistical tests were performed using GraphPad Prism 7 software.

## 3. Results

### 3.1. Biocontrol Efficiency in the Field Experiment

The biocontrol efficiency of the different proportions of the agents on the nematodes showed significant differences. The results showed that treatment 1 (*B. nematocida* B16–*P. chlamydosporia* ZK7 = 1:7) had the most significant effects, reaching 77.5% at 30 days, 62.6% at 60 days, and 44.7% at 90 days. The biocontrol efficiency of *B. nematocida* B16 combined with *P. chlamydosporia* ZK7 was 146.82% higher than that of fungus *P. chlamydosporia* ZK7 alone. Treatments 2, 3, and 5 showed no biocontrol efficiency, and the theoretical biocontrol efficiency was only 10.4%, 3.7%, and 2.6% at 90 days. The biocontrol efficiency of *P. chlamydosporia* ZK7 alone was also poor, with values of 31.4%, 16.4%, and 2.6% at 30, 60, and 90 days, respectively. In addition, the positive control avermectin showed stable effects, maintaining approximately 40% biocontrol efficiency for three months (Table 1). The effects of plant height and biocontrol on the nematodes were similar. The growth rate of treatment 1 was still the highest, reaching 60% and 47.9% at 30 and 60 days, respectively, while the growth rates of the positive control were 79.5% and 33.9%, respectively. Treatment 1 had an obvious promoting effect on the ground weight of the tomato plants, and the growth rate reached 100% and 25% at 30 and 60 days, respectively, similar to that of the positive control (Table 2).

### 3.2. Influences of B. nematocida B16 Supernatant on the Germination of Fungal Spores

The mixture of *B. nematocida B16* supernatant and *P. chlamydosporia ZK7* spore suspension at 1:3 had little effect on the germination rate of the spores as compared to the nutrient-broth control. As compared to the blank control, the effects of the supernatant *B. nematocida* B16 at ratios of 1:5 and 1:10 on the spore germination rate of *P. chlamydosporia* ZK7 were not significantly different. However, at a ratio of 1:1, the germination of the fungal spores was lower than that of the control, which indicated that a high concentration of *B. nematocida B16* supernatant could inhibit the spore germination of *P. chlamydosporia* ZK7 (Table 3).

### 3.3. Influences on Egg Infection of Meloidogyne incognita

In the nematode egg infection experiments, the inhibition rate of *B. nematocida B16* supernatant on the nematode eggs was only 21.14%, and there was no significant difference between the mixed treatment and *P. chlamydosporia ZK7* alone, which indicated that *B. nematocida B16* could not promote the infection rate of *P. chlamydosporia ZK7* on nematode eggs (Table 4).

### 3.4. Colonization of P. chlamydosporia ZK7 in the Pot Experiment

**Figure 1:**
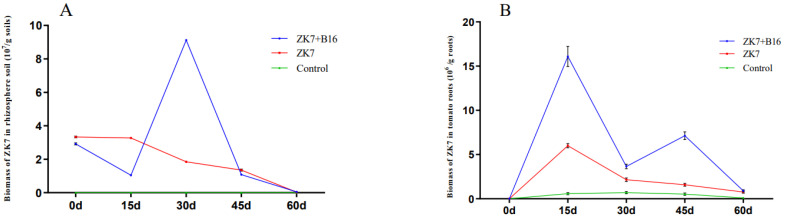
Changes in ZK7 colonization in root and rhizosphere soil of tomato in pot experiment. (**A**): Colonizations of *P. chlamydosporia* ZK7 in tomato rhizosphere soil; (**B**): colonizations of *P. chlamydosporia* ZK7 in tomato root. B16+ZK7: the supernatant of *B. nematocida* B16 was mixed with the spore suspension of *P. chlamydosporia* ZK7 in a ratio of 1:7 (total volume: 100 mL). Data are expressed as the average of three replicates ± SD (standard deviation).

In a separate experiment, the changes in *P. chlamydosporia* ZK7 colonization in the tomato rhizosphere soil gradually decreased in a dose-dependent manner and varied from compound to compound. The colonization of *P. chlamydosporia* ZK7 in the presence of *B. nematocida* B16 reached the maximum value at 30 days, which increased the colonization of fungus *P. chlamydosporia* ZK7 by 392.97%. When *B. nematocida* B16 was mixed with *P. chlamydosporia* ZK7, the change in the colonization of fungus *P. chlamydosporia* ZK7 in the roots showed a gradually increasing trend to the maximum value and then a gradually decreasing trend, reaching a maximum value at 15 days. The colonization of *P. chlamydosporia* ZK7 in the roots increased by 168.17% in the mixed treatment as compared to that in the individual treatment (Figure 1).

### 3.5. Biocontrol Efficiency in the Pot Experiment

Compared with the blank control, the biocontrol efficiency of the *P. chlamydosporia* ZK7 and *B. nematocida* B16 combined treatment was maintained at approximately 50%, and the efficiency of the positive control was 53% and 75% at 30 and 60 days, respectively. Conversely, the biocontrol efficiency of *P. chlamydosporia* ZK7 alone was only 23.1% and 33.4% at 30 and 60 days, respectively (Table 5). At 30 days, the plant height increased compared to that of the control, and the growth rate of *P. chlamydosporia* ZK7 combined with *B. nematocida* B16 reached 31.3%. However, the effect of each treatment on the fresh weight of the aboveground parts was not significantly different. After 60 days, the plant height and fresh weight of the three treatments significantly increased, among which the growth rates of the *P. chlamydosporia* ZK7 and *B. nematocida* B16 combined treatments reached 71.7% and 42.3% for the height and fresh weight, respectively, and the growth rates of *P. chlamydosporia* ZK7 alone reached 9.8% and 19%, respectively. The percentages of the positive controls were 7.8% and 29% for height and fresh weight, respectively (Table 6).

### 3.6. Volatiles Detected by GC/MS

**Figure 2:**
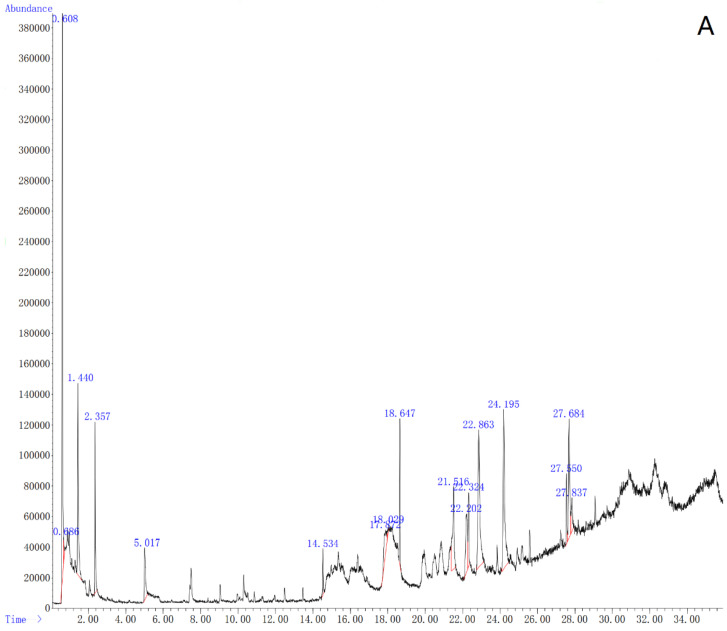

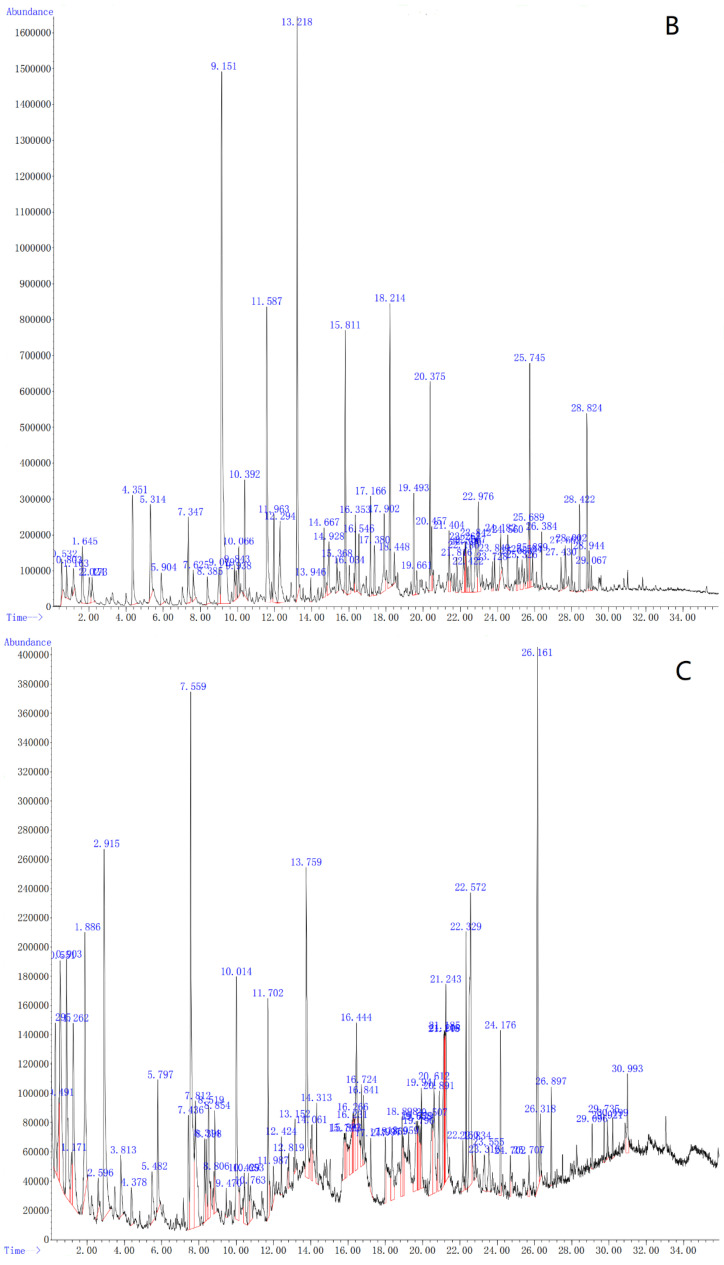
Volatile matters in soil. (**A**) Blank soil samples; (**B**) ZK7; (**C**) ZK7+B16.ZK7+B16: the supernatant of B. *nematocida* B16 was mixed with the spore suspension of P. *chlamydosporia* ZK7 in a ratio of 1:7 (total volume: 100 mL).

Table 7 shows the GC/MS results of *B. nematocida* B16 mixed with *P. chlamydosporia* ZK7 and *P. chlamydosporia* ZK7 alone after controlling for the volatiles of the blank soil samples (Figure 2). By comparing the GC/MS results of the two treatments, the levels of the volatiles furfural, 2-nonanone, hexanal, and (E)-2-hexenal increased, while that of benzaldehyde decreased.

### 3.7. Influences of Volatiles on the Colonization of P. chlamydosporia ZK7

The five VOCs, hexanal, furfural, (E)-2-hexenal, benzaldehyde, and 2-nonanone, promoted the colonization of *P. chlamydosporia* ZK7 in the tomato rhizosphere soil and tomato roots at 200 ppm and 400 ppm, respectively. The enhancement of *P. chlamydosporia* ZK7 colonization in the rhizosphere soil by the five VOCs was 906.55%, 252.41%, 1663.04%, 324.10%, and 1300.3%, respectively. The enhancement of *P. chlamydosporia* ZK7 colonization in the tomato roots was 268.07%, 288.66%, 41.18%, 588.24%, and 155.04%, respectively. At low concentrations, the five VOCs could effectively promote the colonization of *P. chlamydosporia* ZK7, whereas at high concentrations, they could inhibit the colonization of *P. chlamydosporia* ZK7 (Table 8).

## 4. Discussion

The results of the field experiments showed that the biocontrol efficiency of the combination of *P. chlamydosporia* ZK7 and *B. nematocida* B16 was significantly higher than that of *P. chlamydosporia* ZK7 and *B. nematocida* B16 alone. *P. chlamydosporia* ZK7 is a well-studied nematode-egg parasitic fungus. Its mycelium, conidia, and chlamydospores can survive in soil and form infestation filaments to parasitize the eggs and the females of root-knot nematodes [23]. *P. chlamydosporia* ZK7 degrades the nematode body wall and eggshell by producing proteases and chitinases and then kills the larvae and the eggs [24]. *B. nematocida* was found to have nematocidal activity against the nematodes *Panagrellus redivivus* and *Bursaphelenchus xylophilus*, and the mechanisms of attraction and killing were examined in *Caenorhabditis elegans*. *B. nematocida* B16 is eaten by nematodes and secretes serine protease to destroy the intestinal tract of the nematodes [25]. However, the nematocidal activity against *Meloidogyne incognita* may be limited by the stylet and, owing to the complexity and diversity of soil systems, the biocontrol effect of biocontrol fungi in the soil is greatly reduced [4].

Similarly, the addition of *B. nematocida* B16 increased the control effect of fungus *P. chlamydosporia* ZK7 and promoted plant growth. Furthermore, after bacteria were added, two important factors of *P. chlamydosporia* ZK7 that help to control nematodes, spore germination and the egg inhibition rate of *P. chlamydosporia* ZK7, were not affected. However, the colonization of fungus *P. chlamydosporia* ZK7 significantly increased. Bacillus M3–4 can promote the colonization of *Glomus mosseae* and *G. versiforme* around potato roots and can significantly promote the growth of potatoes [26]. An increase in the amount of colonization may be the reason for the increased control effect under the *P. chlamydosporia* ZK7 and *B. nematocida* B16 combination.

In the soil experiment, the rhizosphere colonization of *P. chlamydosporia* ZK7 in the tomato roots increased by 985.03% with the addition of *B. nematocida* B16. Rhizosphere bacteria can promote the colonization of plant roots by fungi [27,28]. When *Pseudomonas aeruginosa* was combined with *P. chlamydosporia* ZK7 to control the tomato root-knot nematodes, the resistance increased by more than 50%, as compared to that under the use of a single agent [10]. When *Fusarium oxysporum* and *Bacillus firmus* were used in combination, their efficacy increased by 58.4% and 22.5%, respectively, as compared to when used alone [29]. The inhibition rate of the root-knot nematodes was significantly improved when *G. mosseae* and *G. versiforme* were used in combination with rhizosphere growth-promoting bacteria (PGPR) [30]. However, the mechanism by which the combination of multiple bacteria improves the control effect remains unclear.

Volatile substances produced by bacteria stimulate mycelial growth and spore production [31]. Auxofuran, a new metabolite isolated from *Streptomyces* AcH505, promotes the growth of mycelia of *Amanita muscaria* at a lower concentration than other metabolites [32]. The interaction between *P. chlamydosporia* ZK7 and *B. nematocida* B16 may be related to certain signaling volatiles. Five kinds of volatile changes in tomato rhizosphere soil after the combination of *B. nematocida* B16 and fungus *P. chlamydosporia* ZK7 were studied. Further analysis and comparison of the five volatiles and the colonization percent of *P. chlamydosporia* ZK7 revealed that the changes in the contents of benzaldehyde and 2-nonanone were the main factors that resulted in an increase in the colonization percent of *P. chlamydosporia* ZK7 in the tomato roots. Furfural is the main volatile substance affecting the colonization of *P. chlamydosporia* ZK7 in the soil.

The five compounds found here have not been reported to enhance the colonization of *P. chlamydosporia* ZK7 or other fungi. Benzaldehyde inhibited the growth of *Aspergillus fumigatus*, *A. terreus*, *A. flavus*, *Cryptococcus neoformans*, and *Candida* spp. [33]. (E)-2-Hexenal inhibited the growth of many pathogenic bacteria, including *Monilinia fructicola* and *Sclerotinia sclerotiorum* [34]. Hexanal showed 100% inhibition of P. expansum and *B. cinerea* conidia at 4.1 mol/L. In addition, it showed significant nematode-killing activity and could significantly improve the plant height and stem diameter of tomato plants within a certain concentration range [35]. In addition, 2-nonanone also inhibited *Alternaria alternata*, *B. cinerea*, and *Colletotrichum gloeosporioides* mycelium growth [36]. Furfural inhibited not only the growth of the bacteria *B. subtilis* and *P. flourescens,* but also the hyphal growth of *Fusarium oxysporum*, *F. solani*, and *Rhizoctonia solani*. The furfural and B. subtilis combination caused a 56.94% reduction in tomato roots [37]. Furfural also effectively killed *M. incognita* [38].

In this study, after the addition of *B. nematocida* B16, the content of volatiles increased in the soil following an increase in the colonization amount of *P. chlamydosporia* ZK7 in the rhizosphere soil and tomato root, resulting in an improved biocontrol efficiency compared to *P. chlamydosporia* ZK7 alone. Furthermore, the increase in hexanal and furfural concentrations in the soil was beneficial not only for the colonization of *P. chlamydosporia* ZK7 but also for the reduction in nematode density. The combined use of *B. nematocida* B16 could improve the control efficiency of *P. chlamydosporia* ZK7 and could be used as an effective method to control root-knot nematode disease.

## 5. Conclusions

In conclusion, our study found that the combined use of *B. nematocida* B16 and *P. chlamydosporia* ZK7 caused volatile ranges that resulted in increased colonization of biocontrol fungi and improved biocontrol efficiency against nematodes. The determination of signal substances by gas chromatography showed that benzaldehyde and 2-nonanone were the main factors that resulted in an increase in the colonization of fungi *P. chlamydosporia* ZK7 in the tomato roots. Furfural was the main volatile substance that affected the colonization of *P. chlamydosporia* ZK7 in the soil. This combined model could be used to promote the ability of biocontrol fungi to control root-knot nematodes.

## Figures and Tables

**Table 1:** Effect of different treatments on galls and biocontrol efficiency in roots of tomato (conducted in field experiments).

Treatment	30 Days		60 Days		90 Days	
	Gall Index	Biocontrol Efficiency (%)	Gall Index	Biocontrol Efficiency (%)	Gall Index	Biocontrol Efficiency (%)
1 (1:7)	1.67 ± 0.43 ***	77.5	2.78 ± 0.68 ***	62.6	4.67 ± 1.55 **	44.7
2 (1:3)	5.92 ± 1.33	20.2	7.25 ± 1.45	14.8	7.56 ± 1.64	10.4
3 (1:2)	6.27 ± 1.73	15.5	7.22 ± 1.89	13.7	8.13 ± 1.26	3.7
4 (ZK7)	5.09 ± 0.25	31.4	6.22 ± 1.17	16.4	8.22 ± 0.77	2.6
5 (B16)	7.00 ± 1.39	5.7	7.44 ± 0.69	8.7	8.22 ± 0.51	2.6
6 (avermectin)	4.42 ± 0.94 *	40.4	4.56 ± 1.20 *	33.9	5.11 ± 0.69 **	39.5
7 (control)	7.42 ± 1.46	-	7.44 ± 1.17	-	8.44 ± 0.38	-

The supernatant of *B. nematocida* B16 was mixed with the spore suspension of *P. chlamydosporia* ZK7 in ratios of 1:7, 1:3, and 1:2 (*v*/*v*); control was sterile deionized water. Data are expressed as the average of three replicates ± SD (standard deviation). * *p* < 0.05, ** *p* < 0.01, *** *p* < 0.001.

**Table 2:** Effects of different treatments on plant height and fresh weight of tomato (conducted in field experiments).

Treatment	30 Days		60 Days	
	Plant Height	Fresh Weight	Plant Height	Fresh Weight
1 (1:7)	31.80 ± 4.00	0.40 ± 0.04 ***	107.25 ± 16.14	0.50 ± 0.02 ***
2 (1:3)	20.80 ± 3.23	0.25 ± 0.03	83.25 ± 26.50	0.58 ± 0.01 ***
3 (1:2)	29.13 ± 7.73	0.35 ± 0.03 ***	82.42 ± 22.20	0.45 ± 0.03
4 (ZK7)	21.87 ± 5.14	0.40 ± 0.02 ***	95.75 ± 34.07	0.52 ± 0.04 ***
5 (B16)	23.33 ± 7.65	0.25 ± 0.02	78.83 ± 35.00	0.38 ± 0.01
6 (avermectin)	35.67 ± 4.21	0.45 ± 0.03 ***	97.08 ± 15.62	0.55 ± 0.03 ***
7 (control)	19.87 ± 5.12	0.20 ± 0.03	72.50 ± 18.11	0.40 ± 0.02

The supernatant of *B. nematocida* B16 was mixed with the spore suspension of *P. chlamydosporia* ZK7 in ratios of 1:7, 1:3, and 1:2 (*v*/*v*); control was sterile deionized water. Data are expressed as the average of three replicates ± SD (standard deviation). *** *p* < 0.001.

**Table 3:** Influences of *B. nematocida* B16 supernatant on germination of fungal spore.

Treatment	Germination of Fungal Spore (%)			
	4 h	8 h	12 h	24 h
1:1	8.81 ± 1.70 ***	33.78 ± 2.05 ***	74.51 ± 3.09 ***	78.07 ± 2.49 **
1:3	23.58 ± 1.25	55.79 ± 3.30	82.42 ± 1.25	85.18 ± 3.56
1:5	30.54 ± 4.71	74.79 ± 0.82	85.56 ± 3.74	89.28 ± 6.55
1:7	41.93 ± 8.58 **	83.16 ± 1.63 ***	93.79 ± 4.97 *	95.08 ± 2.83 *
1:10	26.16 ± 1.63	71.72 ± 3.09	72.80 ± 1.70	86.87 ± 2.49
ZK7	32.78 ± 2.87	66.32 ± 2.16	85.65 ± 3.40	92.88 ± 4.19
NB	22.79 ± 1.89	55.67 ± 1.41	85.86 ± 2.05	87.29 ± 3.09

The supernatant of *B. nematocida B16* was mixed with the spore suspension of *P. chlamydosporia* ZK7 in ratios of 1:1, 1:3, 1:5, 1:7, and 1:10 (*v*/*v*); ZK7 spore suspension was used as blank control; the mixture of NB medium and ZK7 spore suspension 1:3 was used as medium control (total volume: 4 mL). Data are expressed as the average of three replicates ± SD (standard deviation). * *p* < 0.05, ** *p* < 0.01, *** *p* < 0.001.

**Table 4:** Influences of different treatments on eggs of *M*. *incognita* by ZK7.

Treatment	Number of Hatching	Relative Inhibition Rate (%)
B16	134.14 ± 11.00	21.14
ZK7	32.67 ± 9.84	79.28
B16 + ZK7	81.75 ± 11.83	68.16
Control	157.67 ± 11.12	-

“-”: no data. B16 + ZK7: the supernatant of *B. nematocida* B16 was mixed with the spore suspension of *P. chlamydosporia* ZK7 in a ratio of 1:7 (*v*/*v*); control was sterile deionized water. Data are expressed as the average of three replicates ± SD (standard deviation).

**Table 5:** Effect of different treatments on galls and biocontrol efficiency in roots of tomato (collected in pot experiments).

Treatment	30 Days		60 Days	
	Gall Index	Biocontrol Efficiency (%)	Gall Index	Biocontrol Efficiency (%)
ZK7+B16	2.33 ± 1.56	46.2	2.00 ± 0.67	50
ZK7	3.33 ± 0.44	23.1	2.67 ± 0.44	33.4
0.5% Avermectin	2.00 ± 1.33 *	53.8	1.00 ± 0.44 **	75
Control	4.33 ± 0.44	-	4.00 ± 1.33	-

“-”:no data. ZK7+B16: *B*. *nematocida* B16 fermentation broth and *P. chlamydosporia* ZK7 fermentation broth were evenly mixed in a ratio of 1:7 (total volume: 100 mL); control was sterile deionized water. Data are expressed as the average of three replicates ± SD (standard deviation). * *p* < 0.05, ** *p* < 0.01.

**Table 6:** Effects of different treatments on plant height and fresh weight of tomato (collected in pot experiments).

Treatment	30 Days		60 Days	
	Plant Height (cm)	Fresh Weight (g)	Plant Height (cm)	Fresh Weight (g)
ZK7+B16	22.33 ± 4.04	4.45 ± 0.22	56.67 ± 5.51 ***	19.40 ± 6.56
ZK7	18.67 ± 5.51	2.86 ± 0.58	46.00 ± 7.55 *	16.23 ± 4.45
0.5% avermectin	18.33 ± 4.16	2.31 ± 0.67	50.00 ± 7.94 **	17.59 ± 3.12
Control	17.00 ± 3.00	4.39 ± 0.89	33.00 ± 2.64	13.64 ± 3.46

ZK7+B16: *B*. *nematocida* B16 fermentation broth and *P. chlamydosporia* ZK7 fermentation broth were evenly mixed in a ratio of 1:7 (100 mL); control was sterile deionized water. Data are expressed as the average of three replicates ± SD (standard deviation). * *p* < 0.05, ** *p* < 0.01, *** *p* < 0.001.

**Table 7:** Volatile matters in soil.

	Volatiles	Area%	
		B16 + ZK7	ZK7
1	Hexanal	11.17	1.91
2	(E)-2-Hexenal	0.49	0.32
3	Furfural	2.7	0.79
4	Benzaldehyde	8.71	15.89
5	Octanal	1.37	3.46
6	Benzeneacetaldehyde	6.76	6.05
7	2-Nonanone	13.51	6.51
8	2-Undecanone, 6,10-dimethyl-	1.47	2.6
9	Furan, 2-pentyl-	-	1.43
10	Dibenzofuran	-	1.47
11	Fluorene	-	1.66

Note: “-” indicates that VOCs were not detected.

**Table 8:** Colonization of *P. chlamydosporia ZK7* in rhizosphere soil and tomato roots with volatile compounds.

Treatment	Biomass of ZK7 in Soil (10^3^/g Soils)			Biomass of ZK7 in Root (10^3^/g Soils)		
	200 ppm	400 ppm	800 ppm	200 ppm	400 ppm	800 ppm
Hexanal	2474 ± 40 ***	3812 ± 37 ***	8143 ± 43 ***	325 ± 22 *	876 ± 24 ***	367 ± 18 ***
(E)-2-Hexanal	2500 ± 41 ***	2851 ± 29 ***	1109 ± 21 ***	332 ± 24 **	952 ± 26 ***	670 ± 11 ***
Furfural	1746 ± 36 ***	3338 ± 32 ***	14263 ± 176 ***	246 ± 18	336 ± 23 **	341 ± 17 **
Benzaldehyde	1343 ± 25 ***	3431 ± 19 ***	603 ± 11 **	257 ± 21	425 ± 17 ***	1638 ± 42 ***
2-Nonanone	1319 ± 41 ***	11329 ± 154 ***	7110 ± 10 ***	607 ± 27 ***	524 ± 28 ***	321 ± 19 *
Fermentation broth	505 ± 88 ***			1018 ± 72 ***		
Control	809 ± 11			238 ± 16		

Data are expressed as the average of three replicates ± SD (standard deviation). * *p* < 0.05, ** *p* < 0.01, *** *p* < 0.001.

## Data Availability

Not applicable.
